# What could be the role of genetic tests and machine learning of *AXIN2* variant dominance in non-syndromic hypodontia? A case-control study in orthodontically treated patients

**DOI:** 10.1186/s40510-024-00532-4

**Published:** 2024-08-26

**Authors:** Nora Alhazmi, Ali Alaqla, Bader Almuzzaini, Mohammed Aldrees, Ghaida Alnaqa, Farah Almasoud, Omar Aldibasi, Hala Alshamlan

**Affiliations:** 1 Department of Preventive Dental Sciences, College of Dentistry, King Saud bin Abdulaziz University for Health Sciences, King Abdullah International Medical Research Center, Ministry of the National Guard Health Affairs, Riyadh, Saudi Arabia; 2 Department of Restorative and Prosthetic Dental Sciences, College of Dentistry, King Saud bin Abdulaziz University for Health Sciences, King Abdullah International Medical Research Center, Ministry of the National Guard Health Affairs, Riyadh, Saudi Arabia; 3 Department of Medical Genomics Research, King Abdullah International Medical Research Center, King Saud bin Abdulaziz University for Health Sciences, Ministry of the National Guard Health Affairs, Riyadh, Saudi Arabia; 4 College of Dentistry, King Saud bin Abdulaziz University for Health Sciences, King Abdullah International Medical Research Center, Ministry of the National Guard Health Affairs, Riyadh, Saudi Arabia; 5 Biostatistics Section, King Abdullah International Medical Research Center, King Saud bin Abdulaziz University for Health Sciences, Ministry o the National Guard Health Affairs, Riyadh, Saudi Arabia; 6 Department of Information Technology, College of Computer Science, King Saud University, Riyadh, Saudi Arabia

**Keywords:** Machine learning algorithms, Non-syndromic hypodontia, Single nucleotide polymorphism, *AXIN2*, *PAX9*, *MSX1*, Genetics, Orthodontics

## Abstract

**Background:**

Hypodontia is the most prevalent dental anomaly in humans, and is primarily attributed to genetic factors. Although genome-wide association studies (GWAS) have identified single-nucleotide polymorphisms (SNP) associated with hypodontia, genetic risk assessment remains challenging due to population-specific SNP variants. Therefore, we aimed to conducted a genetic analysis and developed a machine-learning-based predictive model to examine the association between previously reported SNPs and hypodontia in the Saudi Arabian population. Our case–control study included 106 participants (aged 8–50 years; 64 females and 42 males), comprising 54 hypodontia cases and 52 controls. We utilized TaqMan^TM^ Real-Time Polymerase Chain Reaction and allelic genotyping to analyze three selected SNPs (*AXIN2*: rs2240308, *PAX9*: rs61754301, and *MSX1*: rs12532) in unstimulated whole saliva samples. The chi-square test, multinomial logistic regression, and machine-learning techniques were used to assess genetic risk by using odds ratios (ORs) for multiple target variables.

**Results:**

Multivariate logistic regression indicated a significant association between homozygous *AXIN2* rs2240308 and the hypodontia phenotype (ORs [95% confidence interval] 2.893 [1.28–6.53]). Machine-learning algorithms revealed that the *AXIN2* homozygous (A/A) genotype is a genetic risk factor for hypodontia of teeth #12, #22, and #35, whereas the *AXIN2* homozygous (G/G) genotype increases the risk for hypodontia of teeth #22, #35, and #45. The *PAX9* homozygous (C/C) genotype is associated with an increased risk for hypodontia of teeth #22 and #35.

**Conclusions:**

Our study confirms a link between *AXIN2* and hypodontia in Saudi orthodontic patients and suggest**s** that combining machine-learning models with SNP analysis of saliva samples can effectively identify individuals with non-syndromic hypodontia.

## Background

Non-syndromic hypodontia is the most prevalent human dental anomaly [1], across various regions and ethnicities [2, 3].The wide variation in prevalence rates (3.48% and 9.4% in the Spanish and Japanese population, respectively) [2, 3] is attributed to differences in the genetic backgrounds, sample sizes, and diagnostic criteria employed in previous studies [4]. In Saudi Arabia, where hypodontia is the predominant dental anomaly, several studies have examined the prevalence of hypodontia in various regions [5]. Regarding congenitally missing teeth, one study reported a 25.7% prevalence [6], whereas another found a prevalence rate of 24.7%, in eastern Saudi Arabia [7]. Furthermore, these disparities in hypodontia prevalence could be ascribed to differences in sample sizes, geographic locations, testing methods, participants’ age groups, and ethnic backgrounds [8].

Dental development is a complex multigenetic process [9]. Hypodontia follows an autosomal dominant pattern of inheritance, with incomplete penetrance and variable expression [10]. Genes associated with non-syndromic hypodontia include *MSX1, PAX9,* and *AXIN2* [11]. *MSX1*, a homeobox gene that modulates epithelial–mesenchymal interactions, crucially modulates early tooth development, with *MSX1* mutations causing failure of tooth development [12]. *PAX9*, a member of the transcription factor family, is associated with autosomal dominant, non-syndromic, and familial hypodontia [13]. *AXIN2*, a Wnt-signaling regulator, is associated with autosomal dominant hypodontia and incisor agenesis [14, 15]. *WNT10* and *SMOC2* mutations cause severe hypodontia [16, 17]. As *PAX9*, *MSX1,* and *AXIN2* are most frequently associated with non-syndromic hypodontia [11], we studied these specific genes.

Machine learning utilizes complex algorithms for healthcare data extraction to enhance clinical effectiveness [18] through models that offer dentists and physicians up-to-date medical knowledge, facilitate optimal patient care, reduce diagnostic and therapeutic errors, and aid health prediction [19, 20]. The identification of genetic predictors of non-syndromic hypodontia for early non-radiographic detection could prevent dental complications, reduce treatment costs, and enhance quality of life [21]. Recognizing hypodontia of permanent teeth facilitates preventive strategies, including primary-teeth preservation and fluoride application [22]. Identification of congenitally missing permanent teeth enables improved case management, including primary-teeth extraction for spontaneous alignment of the remaining teeth and prevention of treatment-related complications [23].

We aimed to establish a model for assessing the genetic risk of non-syndromic hypodontia in the Saudi Arabian population.

## Methods

This case-control study was approved (NRC22R/020/01) by the Institutional Review Board of King Abdullah International Medical Research Center (KAIMRC). This study was performed according to the strengthening the reporting of observational studies in epidemiology (STROBE) guidelines [24].

### Participants

The present study screened the patients who visited the orthodontic clinic of King Abdulaziz Medical City, Riyadh (KAMC-RD) and College of Dentistry (COD), King Saud Bin Abdulaziz University for Health Sciences (KSAU-HS) in Riyadh, Saudi Arabia in December 2021. The participants were randomly selected, enrolled, and categorized into the study and control groups (at least one and no congenitally missing tooth [excluding the third molar], respectively) based on the initial clinical and radiographic examination. The inclusion criteria were: healthy Saudi nationals aged 7–70 years. The exclusion criteria comprised the presence of dental or craniofacial anomalies, craniofacial syndromes, history of jaw trauma, and cardiac, autoimmune, endocrine, bleeding, neurological, kidney, liver, or mental illnesses. By October 2023, we randomly selected a total of 114 participants. All participants underwent a standard clinical and radiographic examination. However, eight of the 114 participants with hypodontia were excluded. Ultimately, we established a case-control study with 54 cases of hypodontia and randomly selected 52 healthy controls from the same source population. The cases and controls were individually matched based on age and sex.

### Clinical assessment

The participants underwent a clinical examination and history-taking. Panoramic radiographs, previously taken for orthodontic treatment, were used to detect and grade hypodontia. Written informed consent was obtained from all participants or their legal guardians. For the scheduled collection of high-quality saliva samples, the participants received written instructions, including refraining from eating, drinking, brushing teeth, chewing gum, or smoking for 1 hour prior to saliva collection.

### Saliva collection and analysis

If the participants followed the specified instructions, the screening and saliva collection visits were combined. Unstimulated whole saliva was collected using the Oragene DNA (OG-500) collection kit (DNA Genotek, Stittsville, Canada) in accordance with the manufacturer’s instructions, and samples were coded to ensure confidentiality; the collection time was recorded, all samples were collected during the same period of the day, and samples were stored at −80°C until analysis.

### DNA extraction

The saliva samples were transported to the medical genomic department at KAIMRC for DNA isolation. DNA was extracted using the prepIT.L2P extraction kit (PT-L2P-5, DNA Genotek, Stittsville, Canada). We assessed DNA quality and quantity with a NanoDrop^TM^ spectrophotometer (Thermo Fisher Scientific, Wilmington, DE, USA) and a Qubit^®^ 3.0 fluorimeter (catalog number: Q33216, Thermo Fisher Scientific, Wilmington, DE, USA), and the examiner (M.A.) who conducted the laboratory analysis was blinded to the group allocation.

### Genotyping and single nucleotide polymorphisms (SNPs) analysis

Genotyping was performed using the TaqMan^TM^ genotyping master mix (Thermo Fisher Scientific, Wilmington, DE, USA) according to the manufacturer's instructions, with a DNA concentration of 20 ng/µL in the final reaction volume of 25 µL. The experiments were conducted on the Applied Biosystems^TM^ QuantStudio^TM^ 6 Flex Real-Time PCR system (Thermo Fisher Scientific, Wilmington, DE, USA). The thermocycler conditions for stages were as follows: pre-read at 60°C for 30 seconds, hold at 95°C for 10 minutes, PCR of 40 cycles at 95°C for 15 seconds and 60°C for 1 minute, and post-read at 60°C for 30 seconds. Genotyping for the selected three SNPs, *AXIN2*: rs2240308, *PAX9*: rs61754301, and *MSX1*: rs12532, was performed using C__26933394_10, C__90244317_10, and C___2577354_1_ assay kits, respectively (Applied Biosystems, Waltham, MA, USA).

### AI-assisted discovery of SNPs

We employed machine learning to perform genetic risk assessment by using odds ratios (OR) for multiple target variables from a dataset ad focusing on teeth #12, #22, #35, and #45, which are the most frequently missing teeth [25–27]. The analysis was implemented and executed using Python programming language and the scikit-learn library.

The logistic regression algorithm from scikit-learn was selected as the machine-learning model because it is commonly employed for binary classification problems and is appropriate for predicting the presence or absence of genetic risks associated with the target variables: teeth #12, #22, #35, and #45. By training individual logistic regression models for each target variable, we sought to delineate the associations between genetic markers (features) and the presence or absence of genetic risk for each specified target.

Prior to model training, the dataset underwent preprocessing. Categorical data, including genetic markers, were transformed into numerical form via one-hot encoding, which enabled the algorithms to accurately interpret and learn from the data. Each categorical marker was converted into several binary columns, wherein each column represented a distinct category or allele.

**Fig. 1:**
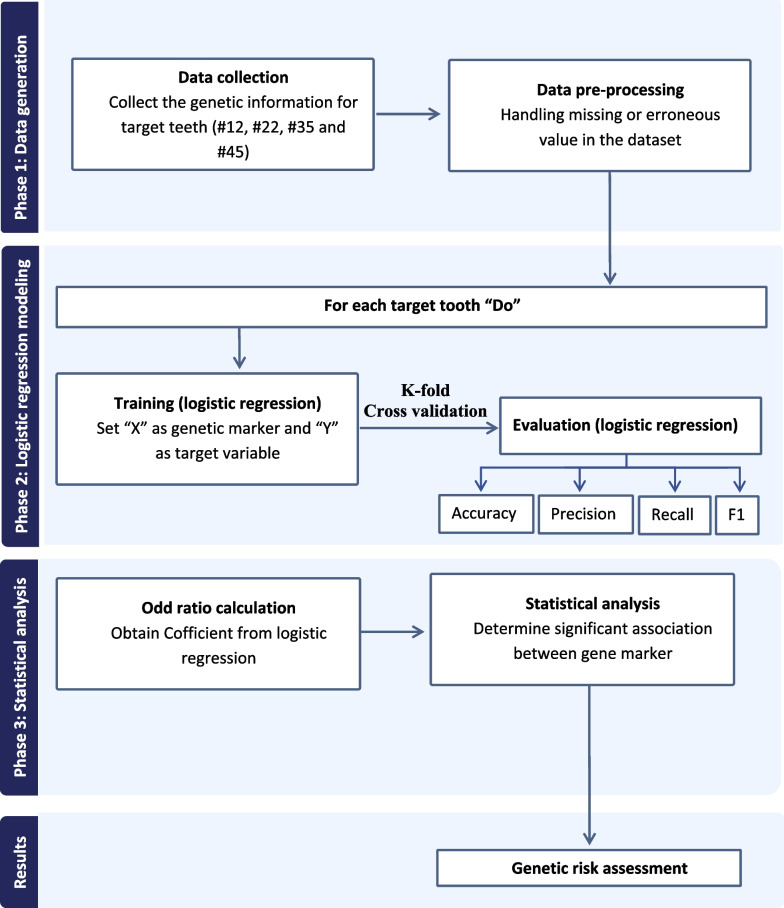
The phases for machine learning model generation. The first phase included data collection and data pre-processing. Phase 2 involved training the model to identify genetic markers and logistic regression was used for evaluation. Phase 3 included statistical analysis using odd ratio calculation and investigation of the association between genetic markers and congenitally missing teeth. The final phase involved the generation of a genetic risk assessment

**Fig. 2:**
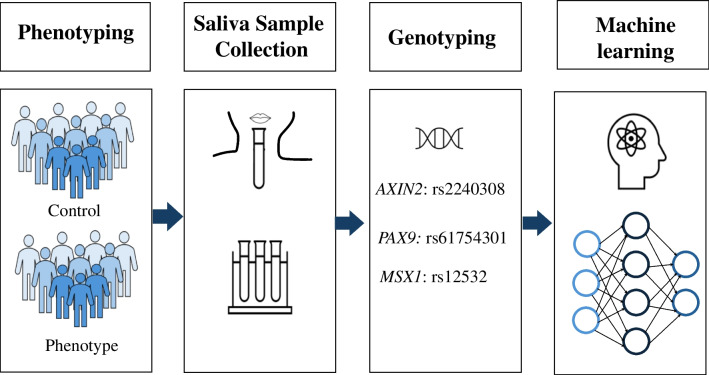
Schematic diagram illustrating the steps conducted to create a machine learning model for genetic risk assessment for hypodontia. The first step in this study was to categorize the participants into control and phenotype groups. Thereafter, unstimulated whole saliva was collected following the manufacturer’s instructions. Then, samples were genotyped. Finally, machine learning algorithms were utilized to conduct a genetic risk assessment model for hypodontia

A logistic regression model was trained on the preprocessed dataset for each target variable. Throughout the training process, the model discerned the relationships between genetic markers and the presence or absence of associated genetic risk for the specific target variable. ORs were computed using the model’s learned coefficients to determine the influence of each genetic marker on the genetic risk assessment of each target variable. An increased or decreased OR signified a more robust or less substantial association, respectively, with the presence of genetic risk. The machine-learning steps are depicted in Fig. 1. The outline of genetic risk assessment of hypodontia is illustrated in Fig. 2.

### Generation of pseudocode

By utilizing a genetic dataset to evaluate dental risk and outputting results for specific teeth, the algorithm assessed genetic risk for dental issues. Data were initially collected from genetic information and the corresponding labels for target teeth (#12, #22, #35, and #45) from individuals who had undergone genetic testing for dental risk assessment. Data preprocessing was undertaken to resolve missing or erroneous values in the dataset and to identify features (genetic markers) and target variables (presence/absence of genetic risk) for each tooth. Logistic regression modeling was conducted for each target tooth by training a model with features (x) as the genetic markers and the target variable (y) as the presence/absence of genetic risk. The model was fitted using these features and target variable, and its performance was evaluated on metrics, such as accuracy, precision, recall, and F1-score. Techniques, such as k-fold cross-validation, were utilized to assess the model’s generalizability. ORs were calculated for each target tooth to derive coefficients from the logistic regression model. Statistical analysis was conducted to determine the significance of associations between gene markers and the risk of dental issues, including hypothesis testing to calculate *P*-values. The algorithm concluded by outputting the genetic risk assessment results for the target teeth.

### Statistical analysis

Based on the Martha et al. study [28], a sample size of 50 participants per group was determined to obtain a Type I error rate of 5% and 90% power from calculations in PASS 2023 version 15 (Power Analysis and Sample Size Software (2023), NCSS, LLC, Kaysville, Utah, USA, nss.com/software/pass).

The clinicodemographic variables were summarized by frequency and proportion or mean and standard deviation (Table 1). Allele frequencies for *MSX1* gene rs12532 (A allele) and *PAX9* gene rs61754301 (C allele) were initially calculated, followed by those for *AXIN2* gene rs2240308 (A allele). Logistic regression served as the primary statistical method to analyze the intergroup differences in the associations between SNPs of *MSX1, PAX9,* and *AXIN2* in the hypodontia and control groups. A backward stepwise-selection approach was employed to construct the final model. The characteristics of the *MSX1*, *PAX9*, and *AXIN2* genes and their alleles are as follows: the *MSX1* (rs12532) gene’s reference allele was A, with G as the alternative allele, resulting in homozygous genotypes of A/A or G/G and heterozygous genotypes of A/G or G/A; the *PAX9* (rs61754301) gene’s reference allele was C, with T as the alternative allele, leading to homozygous genotypes of C/C or T/T and heterozygous genotypes of C/T or T/C; and the *AXIN2* (rs2240308) gene’s reference allele was A, with G as the alternative allele, yielding homozygous genotypes of A/A or G/G and heterozygous genotypes of A/G or G/A.* P*<0.05 were considered statistically significant. All data analyses were performed using SAS version 9.4 (SAS Institute Inc., Cary, NC, USA).

**Table 1:** Characteristics of the studied groups

Groups	n (Percentage)	Female (Percentage)	Male (Percentage)	Age (year) Mean ± Standard deviation
Control	52.00 (49.06%)	32.00 (61.5%)	20.00 (38.5%)	22.83 ± 6.92
Dental agenesis	54.00 (50.94%)	32.00 (59.3%)	22.00 (40.7%)	18.37 ± 8.54
Total/percentage	106.00 (100%)	64.00 (60.4%)	42.00 (39.6%)	20.56 ± 8.07

## Results

The study cohort of 106 Saudi Arabian participants (64 females and 42 males, aged: 8–50 years) was divided into 54 cases with hypodontia and 52 controls. In the study group, the congenital absence of one tooth (53.70%) showed the highest frequency, followed by the absence of two teeth (31.48%). Our findings presented that the most common congenitally missing teeth were #35 and #45 with a prevalence rate of 16.98% among cases, followed by #12 and #22 with a prevalence rate of 14.15%, and 13.20%, respectively.

Table 2 summarizes the intergroup differences in the genotypic distribution and allele frequencies of the three SNPs in the hypodontia and control groups. No statistically significant intergroup difference was observed in the distribution of *MSX1* rs12532, *PAX9* rs61754301, and *AXIN2* rs2240308. Table 3 presents the multivariate logistic regression analysis for genetic risk assessment in the hypodontia group. Individuals in the hypodontia group were more likely to possess a homozygous *AXIN2* rs2240308 (OR [95% confidence interval] 2.893 [1.28–6.53]).

**Table 2:** *MSX1*, *PAX9* and *AXIN2* genotype distribution and allele frequencies

Gene	Polymorphism	Control group (n= 52 )	Hypodontia group (n= 54)	Statistical analysis for the genotype	Statistical analysis for alleles
*MSX1* rs12532	A/G	39.00 (75.00)	34.00 (62.96)	A/G + G/G versus A/A Chi-square=2.09 *P* value=0.148 Odd ratio=0.5182 (0.2112, 1.2713)	G versus A Chi-square=0.86 *P* value=0.353 Odd ratio=1.2966 (0.7477, 2.2484)
	G/A	0.00 (0)	0.00 (0)		
	A/A	10.00 (19.23)	17.00 (31.48)		
	G/G	3.00 (5.77)	3.00 (5.56)		
	A	59.00	68.00		
	G	45.00	40.00		
*PAX9* rs61754301	C/T	25.00 (48.08)	22.00 (40.74)	C/T + T/T versus C/C Chi-square=0.58 *P* value=0.446 Odd ratio=0.7425 (0.3443, 1.6012)	T versus C Chi-square=0.41 *P* value=0.52197 Odd ratio=0.8084 (0.4223, 1.5474)
	T/C	0.00 (0)	0.00 (0)		
	C/C	27.00 (51.92)	32.00 (59.26)		
	T/T	0.00 (0)	0.00 (0)		
	C	79.00	86.00		
	T	25.00	22.00		
*AXIN2* rs2240308	A/G	37.00 (71.15)	28.00 (51.85)	A/G + GG versus A/A *Fisher Exact test* *P* value=0.6207 Odd ratio= 1.0417 (0.2465, 4.4028)	G versus A Chi-square=2.22 *P* value=0.136233 Odd ratio=0.6556 (0.3755, 1.1445)
	G/A	0.00 (0)	0.00 (0)		
	A/A	4.00 (7.69)	4.00 (7.41)		
	G/G	11.00 (21.15)	22.00 (40.74)		
	A	45.00	36.00		
	G	59.00	72.00

**Table 3:** Multivariate logistic regression analysis for hypodontia predictions

Gene	Independent variable	Odds Ratio	95% confidence interval	*P* value
*MSX1* rs12532 (homozygous)	*MSX1* rs12532 (control)	2.242	(0.962, 5.228)	0.0616
*PAX9* rs61754301 (homozygous)	*PAX9* rs61754301 (control)	1.467	(0.679, 3.169)	0.3291
*AXIN 2* rs2240308 (homozygous)	*AXIN 2* rs2240308 (control)	2.893	(1.281, 6.530)	0.0106*

^*^ *P* value < 0.05

Our machine-learning model’s genetic risk assessment for teeth #12, #22, #35, and #45 revealed that, for tooth #12, the *PAX9* homozygous (C/C) genotype was associated with a marginally reduced risk of dental issues as compared to individuals without this allele (OR 0.860989). Conversely, the *PAX9* heterozygous (C/T) genotype conferred a marginally increased risk (OR 1.161477). The *AXIN2* homozygous (A/A) and heterozygous (A/G) genotypes denoted a moderately higher risk (OR 1.364085) or a marginally decreased risk, respectively. The *AXIN2* homozygous (G/G) allele was associated with a slightly lower risk (OR 0.887142).

For tooth #22, the *PAX9* homozygous (C/C) genotype conferred a marginally increased risk of dental issues (OR 1.099051). Conversely, the *PAX9* heterozygous (C/T) genotype exhibited a marginally reduced risk (OR 0.909903). The *AXIN2* homozygous (A/A) and heterozygous (A/G) genotypes conferred a slightly elevated risk (OR 1.040714) and a lower risk (OR 0.7249), respectively. The *AXIN2* homozygous (G/G) genotype created a moderately increased risk (OR 1.325418).

For tooth #35, the *PAX9* homozygous (C/C) genotype was associated with a marginally increased risk (OR 1.034322). Conversely, the *PAX9* heterozygous (C/T) genotype indicated a marginally decreased risk (OR 0.966832). The *AXIN2* homozygous (A/A) and heterozygous (A/G) genotypes signified a slightly elevated risk (OR 1.045664) and a reduced risk (OR 0.817666), respectively. The *AXIN2* homozygous (G/G) genotype denoted a modestly increased risk (OR 1.169603).

For tooth #45, the *PAX9* homozygous (C/C) genotype was associated with a decreased risk (OR 0.689289). Conversely, the *PAX9* heterozygous (C/T) genotype indicated an increased risk (OR 1.450768). The *AXIN2* homozygous (A/A) and heterozygous (A/G) genotypes conferred a decreased risk (OR 0.825193) and a slightly decreased risk (OR 0.847812), respectively. The *AXIN2* homozygous (G/G) genotype was associated with a slightly increased risk (OR 1.429367).

The ORs offer a measure of the association between gene alleles and the risk of dental issues in individual teeth. It is important to note that these associations were relatively modest, with ORs close to 1. Nonetheless, even minor increases or decreases in risk can carry implications for dental health.

## Discussion

In this study, we investigated the use of machine learning to predict hypodontia risk based on selected SNPs in the *MSX1, PAX9,* and *AXIN2* genes. To our knowledge, this is the first orthodontic study to employ a combination of machine learning and hypodontia to thoroughly examine the association between genetic factors and hypodontia.

Machine learning, a subfield of artificial intelligence, has been widely applied to the diagnosis, prediction, and prognosis of various medical conditions and uses statistical algorithms to make decisions or provide data-driven predictions [29]. In this study, we initially examined the genotype distribution and allele frequency of SNPs of the *MSX1, PAX9,* and *AXIN2* genes that were previously identified in GWAS as being associated with hypodontia susceptibility. We conducted multivariate logistic regression analysis to assess the genetic risk for the hypodontia group.

In the present study, the mandibular second premolars were the teeth that were most commonly affected by hypodontia, followed by the maxillary lateral incisors among the patients in the hypodontia group. These findings align with previous research [25–27]; however, our results differ from studies that identified the maxillary lateral incisors as the most frequently congenitally absent teeth [30, 31]. These discrepancies may stem from varied genetic and ethnic backgrounds, as well as underreporting of hypodontia cases. Dahlberg et al. [32] noted that within each tooth class, the key tooth is the most morphologically stable, whereas the others are more variable, prone to reduction, and more frequently missing.

The genes that are most frequently associated with non-syndromic hypodontia are *MSX1*, *PAX9,* and *AXIN2*. SNPs in MSX1, PAX9, and AXIN2 influence the hypodontia phenotype [33]. In this study, we analyzed SNPs previously reported to be associated with non-syndromic hypodontia: *MSX1* rs12532, *PAX9* rs61754301, and *AXIN2* rs2240308 [28]. Our results revealed no significant differences in allele frequency or genotype distribution between the control and hypodontia groups, suggesting that these SNPs may not have influenced hypodontia expression in our cohort. Notably, we combined homozygous variant genotypes with heterozygous genotypes for comparison against wild-type genotypes (Table 3).

Conversely, multivariate logistic regression analysis offers greater power and control over variables and revealed a significant association between *AXIN2* gene variations and non-syndromic hypodontia, corroborating the genetic risk assessment for the hypodontia group. These findings align with those of previous studies [33, 34]. However, our analysis did not show a significant association between *MSX1* and *PAX9* variants and hypodontia, which contrasts with findings from earlier research [28, 33]. This discrepancy may be attributed to differences in the studied populations, and the SNPs tested could be significant specifically in the Saudi Arabian population.

The scikit-learn library in Python provides a comprehensive and efficient set of tools for machine learning methods that comprises various algorithms, including logistic regression, and utilities for data preprocessing, model training, and evaluation. The flexibility and ease of use of this library allowed us to implement genetic risk assessment analysis seamlessly [35]. The ORs obtained from the logistic regression models yielded valuable insights into the association between genetic markers and the presence or absence of genetic risk for each tooth. The presence of the *AXIN2* homozygous (A/A) genotype is a genetic risk factor for hypodontia of teeth #12, #22, and #35. Additionally, the presence of *AXIN2* homozygous (G/G) was a genetic risk factor for hypodontia of teeth #22, #35, and #45. The presence of *PAX9* homozygous (C/C) was a genetic risk factor for hypodontia of teeth #22 and #35. These results contribute to the understanding of genetic factors influencing the risk for specific teeth and can potentially guide personalized dental and healthcare interventions.

With the trend toward more personalized medicine, a phenotype in the oral cavity could serve as a diagnostic marker for systemic health diseases [36]. In addition to the association of hypodontia with syndromes, genetic mutations in tooth formation genes have been linked to other medical conditions, such as cancer [14]. For example, *AXIN2* gene mutations may be implicated in hypodontia along with early-onset colon, prostate, and ovarian cancers [14, 37, 38]. Based on these associations, an orthodontist might refer a patient for clinical and cancer screening. However, it is crucial to note that a recent systematic review reported low-quality evidence of the link between hypodontia and cancer [39].

Considering the constraints of our study, the relatively small sample size may have affected the precision of the associations observed. Consequently, a larger sample size is required to corroborate our findings. Furthermore, our investigation was confined to a limited number of SNPs and preselected genes. Furthermore, we restricted our research to the Saudi Arabian population, and therefore, the results may not be applicable to other populations. Nonetheless, despite these limitations, our data offer important insights into the application of machine learning in the evaluation of the genetic risk of hypodontia.

## Conclusions

Our study identified an association between *AXIN2* and hypodontia in the studied population and highlighting the importance of utilizing machine learning in hypodontia research. Further research with a larger sample size and more SNPs is recommended to explore the validity of machine learning. Additionally, our study could guide orthodontic and dental practices in early genetic diagnosis using noninvasive saliva sampling to facilitate prevention and treatment options for orthodontic patients with hypodontia.

## Appendix 1 STROBE statement—checklist of items that should be included in reports of observational studies

**Table:** 

Section and topic	Item no.	Recommendation	Location where item is reported
Title and abstract	1	(*a*) Indicate the study’s design with a commonly used term in the title or the abstract	Line 3, Page 1
		(*b*) Provide in the abstract an informative and balanced summary of what was done and what was found	Lines 10-25, Pages 1-2
Introduction			
Background/rationale	2	Explain the scientific background and rationale for the investigation being reported	Lines 33-62, Pages 3-4
Objectives	3	State specific objectives, including any prespecified hypotheses	Lines 63-64, Page 4
Methods			
Study design	4	Present key elements of study design early in the paper	Lines 67-70, Page 4
Setting	5	Describe the setting, locations, and relevant dates, including periods of recruitment, exposure, follow-up, and data collection	Lines 72-96, Pages 4-5
Participants	6	(*a*) *Case-control study*—Give the eligibility criteria, and the sources and methods of case ascertainment and control selection. Give the rationale for the choice of cases and controls	Lines 72-96, Pages 4-5
		(*b*) *Case-control study*—For matched studies, give matching criteria and the number of controls per case	Not applicable
Variables	7	Clearly define all outcomes, exposures, predictors, potential confounders, and effect modifiers. Give diagnostic criteria, if applicable	Lines 98-116, Page 5-6
Data sources/ measurement	8	For each variable of interest, give sources of data and details of methods of assessment (measurement). Describe comparability of assessment methods if there is more than one group	Lines 106-116 Page 6
Bias	9	Describe any efforts to address potential sources of bias	Line 104, Page 6
Study size	10	Explain how the study size was arrived at	Lines 160-164 Pages 8
Quantitative variables	11	Explain how quantitative variables were handled in the analyses. If applicable, describe which groupings were chosen and why	Lines 165-179 Page 8
Statistical methods	12	(*a*) Describe all statistical methods, including those used to control for confounding	Lines 165-179 Page 8
		(*b*) Describe any methods used to examine subgroups and interactions	Not applicable
		© Explain how missing data were addressed	Not applicable
		(*d*) *Case-control study*—If applicable, explain how matching of cases and controls was addressed	Not applicable
		© Describe any sensitivity analyses	Not applicable

Results	Item no.	Recommendation	Location where item is reported
Participants	13	(a) Report numbers of individuals at each stage of study—eg numbers potentially eligible, examined for eligibility, confirmed eligible, included in the study, completing follow-up, and analysed	Lines 180-187 Page 9
		(b) Give reasons for non-participation at each stage	Not applicable
		© Consider use of a flow diagram	Not applicable
Descriptive data	14	(a) Give characteristics of study participants (eg demographic, clinical, social) and information on exposures and potential confounders	Lines 72-80 Page 4-5 Line 437 Page 20
		(b) Indicate number of participants with missing data for each variable of interest	Not applicable
		© *Cohort study*—Summarise follow-up time (eg, average and total amount)	Not applicable
Outcome data	15	*Cohort study*—Report numbers of outcome events or summary measures over time	Not applicable
		*Case-control study—*Report numbers in each exposure category, or summary measures of exposure	Lines 188-193 Page 9-10 Line 480, Page 22
		*Cross-sectional study—*Report numbers of outcome events or summary measures	Not applicable
Main results	16	(*a*) Give unadjusted estimates and, if applicable, confounder-adjusted estimates and their precision (eg, 95% confidence interval). Make clear which confounders were adjusted for and why they were included	Lines 218-220 Page 11 Line 457, Page 21
		(*b*) Report category boundaries when continuous variables were categorized	Lines 182-183, Page 9
		© If relevant, consider translating estimates of relative risk into absolute risk for a meaningful time period	Not applicable
Other analyses	17	Report other analyses done—eg analyses of subgroups and interactions, and sensitivity analyses	Not applicable
Discussion			
Key results	18	Summarise key results with reference to study objectives	Lines 224-278 Pages 11-13
Limitations	19	Discuss limitations of the study, taking into account sources of potential bias or imprecision. Discuss both direction and magnitude of any potential bias	Lines 279-285 Page 13-14
Interpretation	20	Give a cautious overall interpretation of results considering objectives, limitations, multiplicity of analyses, results from similar studies, and other relevant evidence	Lines 288-293 Page 14
Generalisability	21	Discuss the eneralizability (external validity) of the study results	Not applicable
Other information			
Funding	22	Give the source of funding and the role of the funders for the present study and, if applicable, for the original study on which the present article is based	Lines 313-315 Page 15

## Data Availability

The datasets used and analyzed in the present study are available from the corresponding author upon reasonable request.

## Abbreviations

*AXIN2*: Axis inhibitor protein 2
COD: College of dentistry
GWAS: Genomic-wide association
KAIMRC: King Abdullah International Medical Research Center
KAMC-RD: King Abdulaziz Medical City in Riyadh
KSAU-HS: King Saud Bin Abdulaziz University for health sciences
*MSX1*: Msh homeobox 1
*PAX9*: Paired box gene 9
Smoc2: *SPARC related modular calcium binding 2*
SNPs: Single nucleotide polymorphisms
STROBE: Strengthening the reporting of observational studies in epidemiology
Wnt10: Wingless-related integration site 10
